# The Essential Role for the RNA Triphosphatase Cet1p in Nuclear Import of the mRNA Capping Enzyme Cet1p-Ceg1p Complex of *Saccharomyces cerevisiae*

**DOI:** 10.1371/journal.pone.0078000

**Published:** 2013-10-30

**Authors:** Naoki Takizawa, Toshinobu Fujiwara, Manabu Yamasaki, Ayako Saito, Akira Fukao, Akio Nomoto, Kiyohisa Mizumoto

**Affiliations:** 1 Laboratory of Basic Biology, Institute of Microbial Chemistry, Tokyo, Japan; 2 Department of Molecular Health Sciences, Graduate School of Pharmaceutical Sciences, Nagoya City University, Nagoya, Japan; 3 Department of Biochemistry, School of Pharmaceutical Sciences, Kitasato University, Tokyo, Japan; Keio University, Japan

## Abstract

mRNA capping is the first cotranscriptional modification of mRNA in the nucleus. In *Saccharomyces cerevisiae*, the first two steps of mRNA capping are catalyzed by the RNA triphosphatase Cet1p and the RNA guanylyltransferase Ceg1p. Cet1p and Ceg1p interact to form a mRNA capping enzyme complex and the guanylyltransferase activity of Ceg1p is stimulated by binding with Cet1p. The Cet1p-Ceg1p complex needs to be transported into the nucleus, where mRNA capping occurs. However, the molecular mechanism of nuclear transport of the Cet1p-Ceg1p complex is not known. Here, we show that Cet1p is responsible and that the Cet1p-Ceg1p interaction is essential for the nuclear localization of the Cet1p-Ceg1p complex. The results indicate that the Cet1p-Ceg1p interaction is important not only for the activation of Ceg1p, but also for nuclear import of the complex.

## Introduction

The m^7^GpppN 5′ cap of eukaryotic messenger RNA (mRNA) is essential for cell viability from yeast to mammals, and is the first cotranscriptional modification of cellular pre-mRNA. The cap structure plays a critical role in mRNA maturation (e.g. polyadenylation and splicing), nuclear export of mRNAs, and efficient translation of the majority of cellular mRNAs (reviewed in [1]–[4]). In addition, 2′-O-methylation of cellular mRNA is important for the discrimination of self and non-self mRNA [5], [6]. The mRNA cap is formed by a series of three essential enzymatic activities (reviewed in [7]–[9]). The first step is the hydrolysis of the γ-phosphate from the 5′-triphosphate end of the nascent transcript by RNA 5′-triphosphatase. The second step is the transfer of the GMP portion of GTP to the diphosphate end of the RNA by RNA guanylyltransferase. The third step is the methylation of the N7 position of the guanine base by RNA (guanine-7)-methyltransferase to produce m^7^GpppN (cap 0). These three enzymes are encoded by separate genes in yeast whereas the first two steps are catalyzed by a single polypeptide chain in metazoans [10]–[15].

Most of the mRNA capping reaction occurs cotranscriptionally, when the nascent pre-mRNA reaches a length of 22–25 nucleotides [16], and is facilitated by the recruitment of mRNA capping enzymes to the site of transcription. RNA guanylyltransferase is known to directly interact with the Ser5-phosphorylated form of the RNA polymerase II (pol II) C-terminal domain (CTD) [17]–[19]. The interaction between RNA guanylyltransferase and Ser5-phosphorylated CTD is conserved from yeast to mammals. Ser5-phosphorylation occurs at the early elongation step of pol II transcription and recruits to the transcription complex several factors required for transcriptional elongation, including RNA guanylyltransferase (Ceg1p) and methyltransferase (Abd1p) in *S. cerevisiae* [19]. The *S. cerevisiae* triphosphatase (Cet1p) is presumed to be recruited to the transcription start site by interaction with Ceg1p and/or other proteins, since the direct interaction of Cet1p with the CTD has not been detected [18], [20].

Cet1p forms a heteromeric complex with Ceg1p, and the Cet1p-Ceg1p interaction is essential for cell viability [21]. The Cet1p-Ceg1p interaction stimulates the guanylyltransferase activity of Ceg1p *in vitro*, stabilizes the Ceg1p against thermal inactivation, and is thought to recruit the Cet1p-Ceg1p complex to the pol II CTD [21]–[23]. Genetic, biochemical and structural analysis reveals that the Cet1p homodimer associates with the Ceg1p C-terminal oligonucleotide binding domain (OB domain) via an extended Cet1p ^247^WAQKW^251^ amino acid motif [22], [24]. The Cet1p WAQKW motif would allow Ceg1p to achieve the conformational changes required for mRNA capping. Mutations of the Cet1p WAQKW motif or the Cet1p-Ceg1p interaction interface of the Ceg1p OB domain lead to either lethality or a temperature-sensitive growth phenotype [24].

Although the enzyme mechanisms of the mRNA capping reaction and the structures of mRNA capping enzymes have been well studied, little is known about the regulation of mRNA capping. Recent studies suggest that cellular factors regulate cap methylation [25]–[29], but it was not revealed whether cellular factors also regulate cap formation. The process of mRNA capping occurs in the nucleus, where Cet1p and Ceg1p are localized [30]. However, it is unknown which protein is responsible for the nuclear transport of the Cet1p-Ceg1p complex. In this study, we reveal the molecular mechanism of nuclear transport of the Cet1p-Ceg1p complex. Our results indicate that Cet1p is responsible for the nuclear localization of the Cet1p-Ceg1p complex, and the interaction between Cet1p and Ceg1p is essential not only for enzymatic activity of Ceg1p, but also for nuclear import of the complex.

## Materials and Methods

### Plasmids

The primers and plasmids used in this paper are listed in Tables S1 and S2. To integrate a GFP fragment to the C-terminus of Cet1 and Ceg1, the GFP-KanMX6 fragment was amplified by PCR from pFA6a-GFP(S65T)-KanMX6. Yeast strains HC201 or HC101 were transformed with the GFP-KanMX6 fragment and selected on SD-G418 plates. The Cet1 promoter-Cet1GFP or Ceg1 promoter-Ceg1GFP fragment was amplified by genomic PCR from the HC201-Cet1GFP strain or the HC101-Ceg1GFP strain, respectively. Each PCR fragment was cloned into pRS313. The Cet1-GFP N-terminal deletion mutant and point mutant plasmids were made by inverted PCR.

### *S. cerevisiae* Strains

The yeast strains used in this paper are listed in Table S3. HC101 and HC201 strains were kindly provided by Dr. Shibagaki (Kitasato University). *mtr10*Δ strain was kindly provided by Dr. E. Hurt (Heidelberg University) [31]. kap104-16 strain was kindly provided by Dr. G. Blobel (The Rockefeller University) [32]. PSY967, PSY1103, PSY1199, and PSY1201 strains were kindly provided by Dr. P. Silver (Harvard Medical School) [33], [34]. Plasmids were introduced into yeast by using the lithium acetate transformation protocol. To make the *cet1*Δ*ceg1*Δ strain, yeast strain HC201 was transformed with a *2 μ URA3 CEG1* plasmid (pYGT6). Then, to knock out *CET1*, the HC201-pYGT6 strain was transformed with a PCR fragment of the KanMX6 cassette from pFA6a-GFP(S65T)-KanMX6, and was selected on a SD-G418 plate. The resulting transformant was spread on a 5-fluoroorotic acid (5-FOA) plate to select *cet1*Δ*ceg1*Δ clones.

### Microscopy

Yeast cells were fixed with ethanol, and DNA was stained with DAPI. Fixed cells were suspended in dH_2_O and the localization of GFP-fused protein was examined by fluorescence microscopy.

### GST Pull-down Assay

Ceg1p OB domain was expressed in *E. coli* as GST-tagged fusions and purified from soluble lysates by glutathione-Sepharose column chromatography. Cet1p, Cet1(4A)p, and Cet1(228–549)p were translated in rabbit reticulocyte lysates with [^35^S]-methionine. After nuclease treatment of the translated reticulocyte lysate, GST pull-down assays were performed in TNE buffer (20 mM Tris-HCl (pH 7.5), 150 mM NaCl, 2 mM EDTA, 1% NP-40).

### Triphosphatase Assay

RNA triphosphatase activity was measured as described previously [35]. Briefly, the reaction mixtures (10 µL) containing 50 mM Tris-HCl (pH 7.9), 0.5 mM MgCl_2_, 0.2 µM [γ-^32^P]-terminated poly(A) as a substrate, and Cet1p or Cet1(4A)p at various concentrations, were incubated for 30 min at 30°C. After incubation, the reaction products were analyzed by polyethyleneimine (PEI) cellulose thin-layer chromatography (TLC) with 0.5 M potassium phosphate buffer (pH 3.4), and the TLC plate was exposed to an imaging plate and visualized using a Typhoon imager.

## Results

### Ceg1p-GFP Accumulated in the Nucleus only when Cet1p-GFP was Co-expresssed in the *cet1*Δ*ceg1*Δ Strain

**Figure 1:**
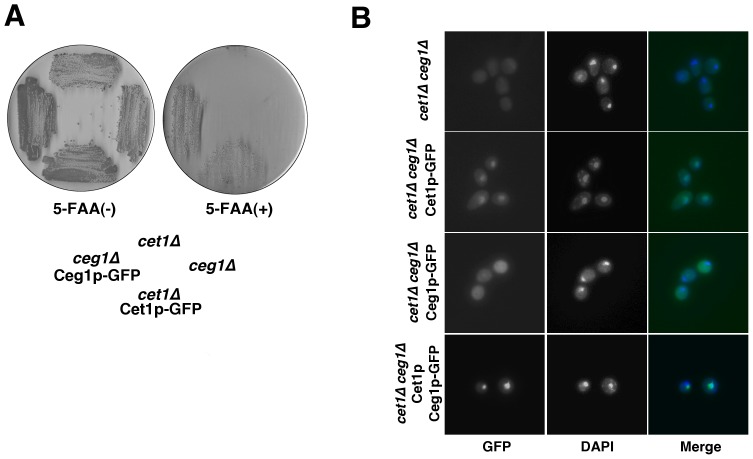
Nuclear accumulation of Cet1p-GFP in the *cet1* Δ***ceg1***Δ **strain.** (A) Complementation of *cet1*Δ and *ceg1*Δ by Cet1p-GFP and Ceg1p-GFP, respectively. Yeast strains HC101 and HC201 were transformed with a *CEN HIS3 CET1-GFP* and *CEN HIS3 CEG1-GFP* plasmid, respectively. His^+^ isolates and control strains were streaked on agar plates with or without 0.075% 5-FAA. These plates were incubated at 30°C for 2 days. (B) Localization of Cet1p-GFP and Ceg1p-GFP in the *cet1*Δ*ceg1*Δ strain. Yeast strain *cet1*Δ*ceg1*Δ was transformed with *CEN HIS3 CET1-GFP*, *CEN HIS3 CEG1-GFP*, or both *2 μ URA3 CET1* and *CEN HIS3 CEG1-GFP* plasmids. The yeast strains were grown in YPD medium and fixed by ethanol. The cell nucleus was stained with DAPI after fixation.

As the capping reaction is catalyzed by the Cet1p-Ceg1p complex in the nucleus, we determined which protein was responsible for nuclear import of the Cet1p-Ceg1p complex. To address this question, the subcellular localization of Cet1p-GFP and Ceg1p-GFP in the *cet1*Δ*ceg1*Δ strain was examined. First, to determine whether Cet1p-GFP and Ceg1p-GFP were functional, plasmid shuffle for complementation of *cet1*Δ and *ceg1*Δ by *CET1-GFP* and *CEG1-GFP*, respectively, was performed. HC101 (*cet1*Δ) and HC201 (*ceg1*Δ) strains were maintained by a human mRNA capping enzyme, hCAP1, on a *CEN TRP1* plasmid under the *ADC1* promoter, because both Cet1p and Ceg1p are essential for yeast cell growth. *Cet1-GFP* and *Ceg1-GFP* were cloned into a *CEN HIS3* plasmid (pRS313) under the control of the *CET1* and *CEG1* native promoters, respectively. HC101 and HC201 were transformed with these plasmids. These transformants and control strains were streaked on YPD plates with 5-fluoroanthranilic acid (5-FAA) to select clones without the *CEN TRP1 hCAP1* plasmid [36]. These transformants are able to grow on agar medium containing 5-FAA if they are transformed with a biologically active *CET1* and *CEG1* allele, respectively. The growth of cells on 5-FAA was complemented by *CET1-GFP* in HC101 and *CEG1-GFP* in HC201 (Figure 1A). This result indicates that Cet1p-GFP and Ceg1p-GFP are functional in the cells.

Next, the subcellular localization of Cet1p-GFP and Ceg1p-GFP in the *cet1*Δ*ceg1*Δ strain was determined. Yeast strain *cet1*Δ*ceg1*Δ was transformed with a *CEN HIS3 CET1-GFP* or a *CEN HIS3 CEG1-GFP* plasmid, and the localization of Cet1p-GFP and Ceg1p-GFP was examined. Cet1p-GFP accumulated in the nucleus but Ceg1p-GFP did not (Figure 1B). When the *cet1*Δ*ceg1*Δ strain was transformed with both *CEN HIS3 CEG1-GFP* and *2 μ URA3 CET1* plasmids, Ceg1p-GFP accumulated in the nucleus (Figure 1B). These results suggest that Cet1p is responsible for nuclear localization of the Cet1p-Ceg1p complex.

### The Cet1p-Ceg1p Interaction, but not the Triphosphatase Activity of Cet1p, is Important for Nuclear Localization of the Cet1p-Ceg1p Complex

**Figure 2:**
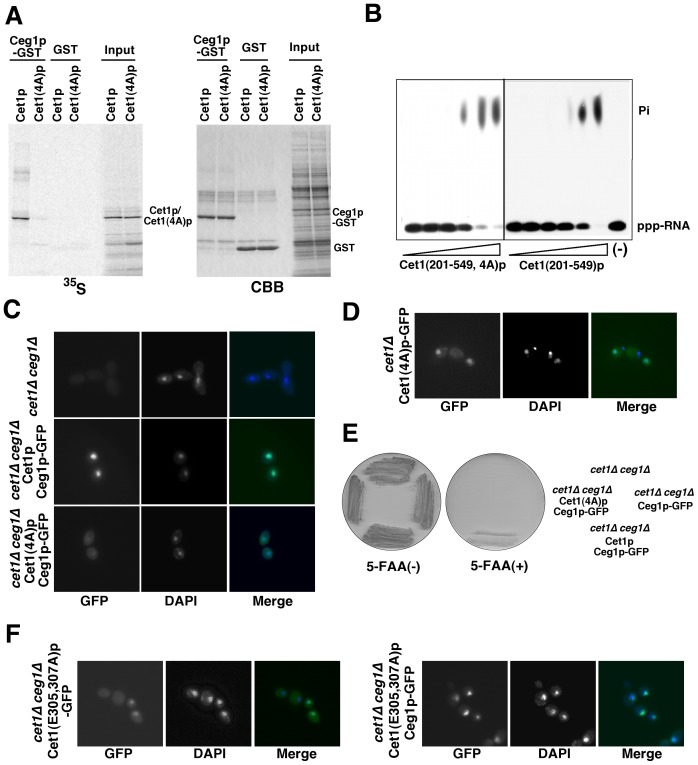
Importance of the Cet1p-Ceg1p interaction for nuclear localization of the Cet1p-Ceg1p complex. (A) GST pull-down assay. A GST pull-down assay was performed using GST (or GST-Ceg1p OB domain) and Cet1p (or Cet1(4A)p) translated in rabbit reticulocyte lysate with [^35^S]-methionine. GST pull-downs were separated by SDS-PAGE and detected with an imaging plate (left) and CBB staining (right). (B) Triphosphatase activity of Cet1(4A)p. The reaction was performed as described in Materials and Methods using [γ-^32^P]-terminated poly(A), and either 2.5, 5, 10, 20, 40, 80 nM Cet1(4A)p or Cet1p. The reaction products were analyzed by PEI-cellulose TLC. The autoradiogram of the thin-layer plate is shown. (C) Localization of Ceg1p-GFP in cells expressing Cet1(4A)p. The yeast strain *cet1*Δ*ceg1*Δ was transformed with both *2 μ URA3 CET1* or *CET1(4A)* and *CEN HIS3 CEG1-GFP* plasmids. The cell nucleus was stained with DAPI. (D) Localization of Cet1(4A)p-GFP. The yeast strain *cet1*Δ*ceg1*Δ was transformed with both *CEN HIS3 CET1(4A)-GFP* and *2 μ URA3 CEG1* plasmids. The cell nucleus was stained with DAPI. (E) Lethal phenotype of the yeast strain expressing Cet1(4A)p and Ceg1p-GFP. The yeast strain used in (C) and control strains were streaked on agar plates with or without 0.075% 5-FAA. These plates were incubated at 30°C for 2 days. (F) Localization of Ceg1p-GFP in cells expressing Cet1p triphosphatase active site mutant. The yeast strain *cet1*Δ*ceg1*Δ was transformed with *CEN HIS3 CET1(E305,307A)-GFP* or both *2 μ URA3 CET1(E305,307A)* and *CEN HIS3 CEG1-GFP* plasmids. The cell nucleus was stained with DAPI.

Next, we tested whether the Cet1p-Ceg1p interaction or the triphosphatase activity of Cet1p is important for nuclear localization of the Cet1p-Ceg1p complex. For this purpose, we utilized Cet1p with a 4x alanine-cluster mutation of ^247^WAQKW^251^ motif (WAQKW to AAAAA, Cet1(4A)p), which has been shown to be essential for the Cet1p-Ceg1p interaction [22]. Cet1(4A)p is supposed to have full triphosphatase activity because Cet1(276–549)p, which dose not include the WAQKW motif, retains triphosphatase activity [37]. Using this mutant, we investigated whether the Cet1p-Ceg1p interaction is necessary for nuclear localization of Ceg1p. To test the interaction between Cet1(4A)p and Ceg1p, a GST pull-down assay was performed using recombinant GST-Ceg1p and Cet1p or Cet1(4A)p translated in rabbit reticulocyte lysate with [^35^S]-methionine. Cet1(4A)p did not copurifiy with GST-Ceg1p, whereas Cet1p copurified with GST-Ceg1p, but not with the negative control, GST (Figure 2A). This result shows that Cet1(4A)p does not interact with Ceg1p. To confirm the RNA-triphosphatase activity of Cet1(4A)p, an *in vitro* assay was performed using recombinant Cet1(201–549)p and Cet1(201–549, 4A)p with 5′ [^32^P]-labeled poly(A) RNA as the substrate. The triphosphatase activity of Cet1(201–549, 4A)p was comparable to that of Cet1(201–549)p, indicating that Cet1(4A)p retains triphosphatase activity *in vitro* (Figure 2B). Next, to determine Ceg1p localization in cells expressing Cet1(4A)p, the yeast strain Δ*cet1*Δ*ceg1* was transformed with both *2 μ URA3 CET1(4A)* and *CEN HIS3 CEG1-GFP* plasmids, and the localization of Ceg1p-GFP was examined. Ceg1p-GFP accumulated in the nucleus when Cet1p was co-expressed, whereas accumulation of Ceg1p-GFP in the nucleus was not observed when Cet1(4A)p was co-expressed (Figure 2C). To address the localization of Cet1(4A)p, the yeast strain HC101 was transformed with a *CEN HIS3 CET1(4A)-GFP* plasmid, and the localization of Cet1(4A)p-GFP was examined. Cet1(4A)p-GFP accumulated in the nucleus (Figure 2D). The ability of Cet1(4A)p to accumulate in the nucleus by itself and its RNA 5′-triphosphatase activity are almost comparable to those of wild-type Cet1p. To determine whether the triphosphatase activity of Cet1p is involved in nuclear localization of the Cet1p-Ceg1p complex, the localization of Cet1p triphosphatase mutant, Cet1(E305,307A)p-GFP, was examined [38]. Cet1(E305,307A)p-GFP accumulated in the nucleus in a background of *cet1*Δ*ceg1*Δ strain (Figure 2F). When the *cet1*Δ*ceg1*Δ strain was transformed with both *CEN HIS3 CEG1-GFP* and *2 μ URA3 CET1(E305,307A)* plasmids, Ceg1p-GFP accumulated in the nucleus (Figure 2F). These results suggest that the triphosphatase activity of Cet1p is not involved in nuclear localization of the Cet1p-Ceg1p complex. Taken as a whole, the binding ability of Cet1p to Ceg1p, but not the RNA 5′-triphosphatase activity of Cet1p, is necessary for nuclear localization of the Cet1p-Ceg1p complex.

To determine the viability of yeast cells expressing Cet1(4A)p, plasmid shuffle for complementation of *cet1*Δ by Cet1p or Cet1(4A)p was performed. Yeast strains *cet1*Δ*ceg1*Δ, harboring the *2 μ URA3 CET1* and *CEN HIS3 CEG1-GFP* plasmids, and *cet1*Δ*ceg1*Δ, harboring the *2 μ URA3 CET1(4A*) and *CEN HIS3 CEG1-GFP* plasmids, were streaked on YPD plates with or without 5-FAA. Growth of the strain on 5-FAA was complemented by *CET1*, but not by *CET1(4A)* (Figure 2E). This result suggests that the Cet1p-Ceg1p interaction is essential for yeast cell growth. To determine whether nuclear localization of Cet1 and Ceg1 is sufficient for cell growth, plasmid shuffle for complementation of *cet1*Δ*ceg1*Δ was employed by expressing Cet1(4A)p and Ceg1p-GFP fused with the SV40 nuclear localization signal (NLS-Ceg1p-GFP). NLS-Ceg1p-GFP was localized in the nucleus when Cet1(4A)p was co-expressed (Figure S1A). However, the expression of both Cet1(4A)p and NLS-Ceg1p-GFP could not rescue growth of the *cet1*Δ*ceg1*Δ strain (Figure S1B). It is important to note that the functional interaction between Cet1p and Ceg1p is essential for yeast cell growth, while nuclear localization of both proteins without their interaction is not sufficient.

### The Cet1p 223–549 Region is Sufficient for Nuclear Localization of Cet1p

**Figure 3:**
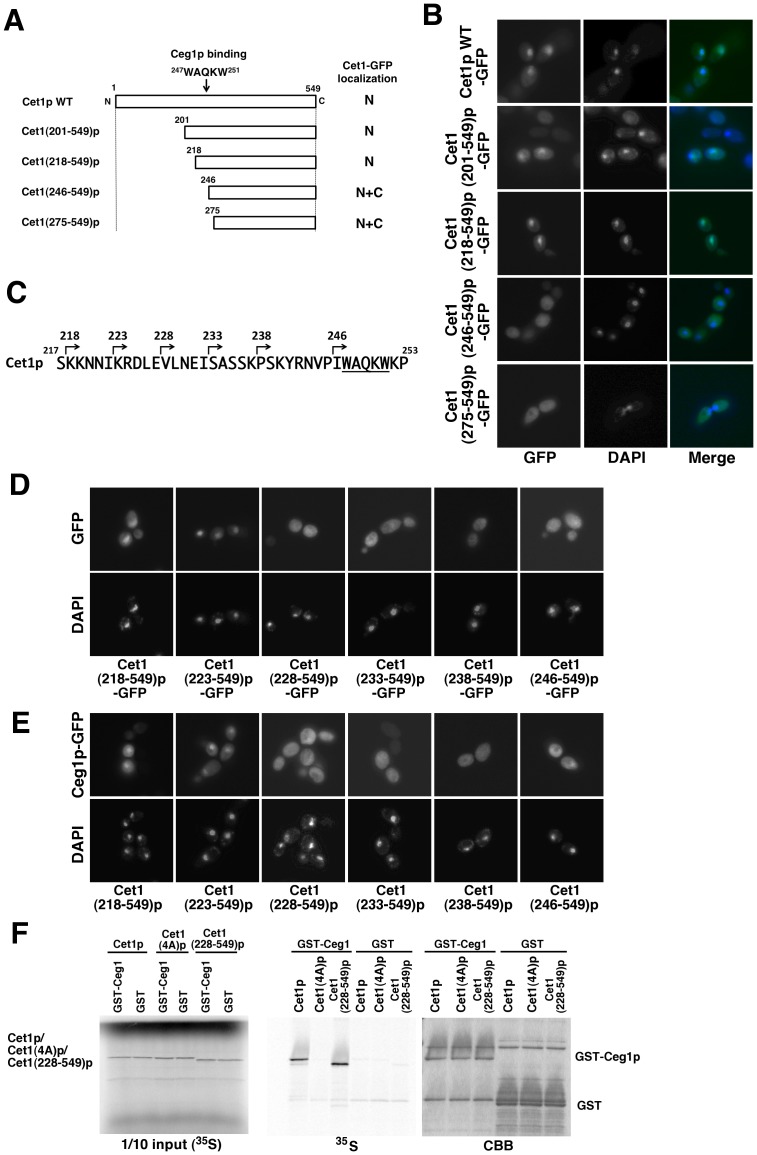
Characterization of the amino acid sequence required for nuclear localization of the Cet1p-Ceg1p complex. (A) Cet1p N-terminal deletion mutants. Localization results shown in (B) are summarized. N: localized in the nucleus. N+C: localized in both the nucleus and the cytoplasm. (B) Localization of Cet1p-GFP mutants in the *cet1*Δ*ceg1*Δ strain. The yeast strain *cet1*Δ*ceg1*Δ was transformed with the *CEN HIS3 CET1-GFP* N-terminal deletion mutant plasmid indicated in (A). The cell nucleus was stained with DAPI. (C) Amino acid sequence between residues 217 and 253 of Cet1p and Cet1p N-terminal deletion mutants. The underline indicates the WAQKW motif for Ceg1p binding. (D) Localization of the Cet1p-GFP N-terminal truncated mutants. The yeast strain *cet1*Δ*ceg1*Δ was transformed with the *CEN HIS3 CET1-GFP* N-terminal deletion mutant plasmid indicated in (C). The cell nucleus was stained with DAPI. (E) Localization of Ceg1p-GFP in cells expressing Cet1p N-terminal deletion mutants. The yeast strain *cet1*Δ*ceg1*Δ was transformed with both a *2 μ URA3 CET1* N-terminal deletion mutant plasmid and the *CEN HIS3 CEG1-GFP* plasmid. The cell nucleus was stained with DAPI. (F) Interaction between His-Cet1(228–549)p and GST-Ceg1. GST pull-down assay was performed using GST or GST-Ceg1p OB domain and Cet1p, Cet1(4A)p, or Cet1(228–549)p translated in rabbit reticulocyte lysate with [^35^S]-methionine. GST pull-downs were separated by SDS-PAGE and detected with an imaging plate (left) and CBB staining (right).

To characterize the region of Cet1p responsible for nuclear localization of the Cet1p-Ceg1p complex, the following series of N-terminal deletion mutants was generated: *Cet1(201*–*549)*, *Cet1(218*–*549)*, *Cet1(246*–*549)*, and *Cet1(275*–*549)* (Figure 3A). These C-terminal GFP-fused Cet1p mutant genes were cloned into a *CEN HIS3* plasmid under the control of the *CET1* promoter. When yeast strain *cet1*Δ*ceg1*Δ was transformed with these plasmids, Cet1p-GFP, Cet1(201–549)p-GFP, and Cet1(218–549)p-GFP accumulated in the nucleus, but Cet1(246–549)p-GFP and Cet1(275–549)p-GFP were localized in both the nucleus and cytoplasm (Figure 3B). These results suggest that the Cet1p 218–549 region is sufficient for nuclear accumulation of Cet1p. To determine the region of Cet1p required for its nuclear accumulation in more detail, the following series of N-terminal deletion mutants were generated: *Cet1(223*–*549)*, *Cet1(228*–*549)*, *Cet1(233*–*549)*, and *Cet1(238*–*549)* (Figure 3C). These C-terminal GFP-fused mutant genes were cloned into a *CEN HIS3* plasmid under control of the *CET1* promoter. When yeast strain *cet1*Δ*ceg1*Δ was transformed with these plasmids, Cet1(223–549)p-GFP accumulated in the nucleus, but Cet1(228–549)p-GFP, Cet1(233–549)p-GFP, and Cet1(238–549)p-GFP did not (Figure 3D). These results suggest that the Cet1p 223–549 region is sufficient for nuclear accumulation of Cet1p.

To analyze the mechanism of Cet1p nuclear import more detail, the localization of mutant Cet1p with residues between position 223 and 227 replaced with alanines was examined. Cet1(223–549, 223–227A)p-GFP did not accumulate in the nucleus (Figure S2A). However, Cet1(223–227A)p-GFP accumulated in the nucleus (Figure S2A). To determine the Cet1p nuclear import pathway, the localization of Cet1p-GFP in *kap* mutant strains was examined. Cet1p-GFP accumulated in the nucleus in all *kap* mutant strains (Figure S2B). These results suggest that Cet1p has more than one nuclear localization signal and is transported into the nucleus via more than one pathway.

Next, to confirm the localization of Ceg1p in cells expressing Cet1p N-terminal deletion mutants, the yeast strain *cet1*Δ*ceg1*Δ was transformed with both a *CEN HIS3 CEG1-GFP* plasmid and a *2 μ URA3 CET1(223*–*549), CET1(228*–*549), CET1(233*–*549),* or *CET1(238*–*549)* plasmid, and the localization of Ceg1p-GFP was examined. Ceg1p-GFP accumulated in the nucleus when Cet1(218–549)p or Cet1(223–549)p was co-expressed, but not when Cet1(228–549)p, Cet1(233–549)p, Cet1(238–549)p, or Cet1(246–549)p was co-expressed (Figure 3E). These results confirm that the localization of Ceg1p is determined by Cet1p. Moreover, Cet1(228–549)p binds to Ceg1p *in vitro* (Figure 3F). Cet1(228–549)p could stabilize Ceg1p and Ceg1p-GFP did not accumulate in the nucleus when Cet1(228–549)p was co-expressed. These results suggest that stability of Ceg1p does not affect localization of Ceg1p.

### The Amount of Nuclear Cet1p-Ceg1p Complex is a Rate-limiting Factor for Yeast Cell Growth

**Figure 4:**
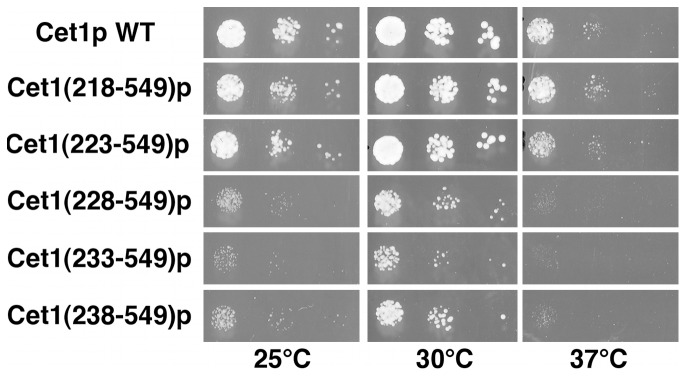
Slow-growth phenotype of the Cet1p-Ceg1p mislocalization strains. The yeast strain HC101, harboring *CEN HIS3 CET1-GFP* N-terminal deletion mutants (without *CEN TRP1 hCAP1*) were grown in YPD medium and put on agar plates. These plates were incubated at 25, 30, or 37°C for 2 days.

Next, we tested whether nuclear accumulation of the Cet1p-Ceg1p complex affects yeast cell growth. To do this, the yeast strain HC101 was transformed with *CEN HIS3 CET1(218*–*549)-GFP, CET1(223*–*549)-GFP, CET1(228*–*549)-GFP, CET1(233*–*549)-GFP, CET1(238*–*549)-GFP,* or *CET1(246*–*549)-GFP,* and these strains were streaked on YPD plates containing 5-FAA to select the clones without the *CEN TRP1 hCAP1* plasmid. The *CEN HIS3 CET1(246*–*549)-GFP* transformant was unable to grow on 5-FAA at any temperature (data not shown). The other 5 transformants were able to grow on 5-FAA at 30°C. To characterize the growth of these transformants, 5-FAA-resistant clones were grown and put on YPD plates at 25, 30, and 37°C, in parallel with *CET1*. The growth of the strains expressing Cet1(218–549)p and Cet1(223–549)p was comparable to that expressing Cet1p at any temperature but the strains expressing Cet1(228–549)p, Cet1(233–549)p, and Cet1(238–549)p grew more slowly than the strain expressing Cet1p at any temperature (Figure 4). This result is consistent with the results of nuclear localization of these mutant proteins (Figures 3D and 3E). These results suggest that mislocalization of the Cet1p-Ceg1p complex leads to a slow-growth phenotype. The small amount of nuclear Cet1p-Ceg1p complex is expected to result in a slow-growth phenotype, because these Cet1p mutants were localized to both the cytoplasm and nucleus. To determine if this is the case, these Cet1p mutants were expressed by a high copy plasmid. The growth of yeast cells expressing the Cet1p mutants was comparable to that expressing Cet1p (Figure S3). These results indicate that the amount of mRNA capping enzyme in the nucleus is an important determinant of yeast cell growth rate.

## Discussion

The regulation of nuclear transport of mRNA capping enzymes is important for mRNA capping, because mRNA capping occurs in the nucleus. Here, we demonstrate that Cet1p is responsible for the nuclear transport of the Cet1p-Ceg1p capping enzyme complex (Figure 1B) and the interaction of Ceg1p with Cet1p is necessary for nuclear transport of the complex (Figure 2C). Furthermore, we show that mislocalization of the Cet1p-Ceg1p complex induces a slow-growth phenotype (Figures 3D, 3E, and 4).

Cet1(223–549)p-GFP accumulated in the nucleus, but Cet1(228–549)p-GFP and Cet1(223–549, 223–227A)p-GFP did not (Figures 3D and S2A). However, Cet1(223–227A)p-GFP accumulated in the nucleus and Cet1p-GFP accumulated in the nucleus at *kap* mutant strains (Figure S2A and S2B). Cet1p would be transported into the nucleus via more than one pathway. The conventional NLS sequences are not found in Cet1. It is possible that Cet1p is transported into the nucleus by the other protein that binds to Cet1p 1–227 region. Homodimerization of Cet1p is important for *in vivo* function of Cet1p, but the role of the homodimerization is not known [37], [39], [40]. Cet1(228–549)p-GFP which did not accumulate in the nucleus could form a homodimer because Cet1(231–549)p form a homodimer [37]. Thus, homodimerization of Cet1p would not be involved in the nuclear import of Cet1p-Ceg1p complex, but the possibility that Cet1p homodimer stabilizes the interaction between Cet1p and nuclear import cargo proteins is still not excluded.

Ceg1p and the Cet1(228–549)p mutant did not accumulate into the nucleus but small amounts of the proteins were still localized to the nucleus (Figures 1B and 3D). GFP-fused Ceg1p and Cet1p mutants used in this study were too large to pass through the nuclear pore complex by passive diffusion. Thus, Ceg1p and Cet1(228–549)p itself could be weakly transported into the nucleus. However, this weak transport is not sufficient for vegetative yeast cell growth, because the strains expressing Cet1(228–549)p grew more slowly than the strain expressing the Cet1(223–549)p which accumulated in the nucleus (Figure 4).

Prior studies suggest that Cet1p-Ceg1p interaction stimulates the guanylyltransferase activity of Ceg1p, stabilizes the Ceg1p against thermal inactivation, and recruit the Cet1p-Ceg1p complex to the pol II CTD [21]–[23]. The amount of Ceg1p-GFP derived from pRS313-Ceg1 GFP plasmid in *cet1*Δ*ceg1*Δ strain is lower than that in HC201 (*ceg1*Δ) strain (data not shown). This result suggests that Cet1p stabilizes the Ceg1p *in vivo*, and gives rise to the possibility that mislocalization of Ceg1p could be attributed to unstabilized Ceg1p. However, Cet1(228–549)p copurified with GST-Ceg1p and Ceg1p-GFP did not accumulate into the nucleus with Cet1(228–549)p (Figures 3E and 3F). This means that Cet1(228–549)p would stabilize Ceg1p-GFP but mislocalization of Ceg1p occurs. Our results indicate that the stabilization of Ceg1p by binding to Cet1p is not involved in the localization of Ceg1p. After nuclear import of the Cet1p-Ceg1p complex, the mRNA capping enzyme is recruited to the transcription start site by binding Ceg1p to pol II CTD [18]. The interaction between the mRNA capping enzyme and pol II CTD is presumed to be important for nuclear retention of the mRNA capping enzyme. However, Cet1p accumulated in the nucleus without Ceg1p (Figure 1). This result indicates that the binding of Ceg1p to pol II CTD is not necessary for nuclear localization of the Cet1p-Ceg1p complex. Takase et al. reported that Cet1p is recruited to the pol II transcription complex by a mechanism other than binding to guanylyltransferase [20]. Thus, other proteins might be involved in the transport and retention of Cet1p into the nucleus by binding to Cet1p.

Expression of Cet1(228–549)p, Cet1(233–549)p, and Cet1(238–549)p in the HC101 strain led to a slow-growth phenotype whereas expression of Cet1(246–549)p could not rescue the *cet1*Δ strain (Figure 4 and data not shown). Although Cet1(246–549)p has the ^247^WAQKW^251^ Ceg1p binding domain and triphosphatase activity, the binding between Cet1(246–549)p and Ceg1p is somehow different from that of Cet1p-Ceg1p [37]. Thus, expression of Cet1(246–549)p could not rescue the *cet1*Δ strain. We note that the strains expressing Cet1(228–549)p, Cet1(233–549)p, and Cet1(238–549)p showed both a slow-growth and a temperature-sensitive phenotype (Figure 4). The transcription of stress genes could be inhibited, because the amount of nuclear mRNA capping enzyme might not be enough for stress response transcription.

The growth of cells expressing Cet1(228–549)p, Cet1(233–549)p, Cet1(238–549)p, and Cet1(249–549)p from a high copy plasmid was comparable to that of the wild-type strain (Figure S3). Although these Cet1p mutants did not accumulate in the nucleus, the amount of nuclear Cet1p was enough for vegetative growth when Cet1p mutants were expressed from a high copy plasmid. These findings raise the hypothesis that the amount of nuclear mRNA capping enzyme could regulate yeast cell growth; however, evidence for this hypothesis has not been found thus far. It is still possible that gene expression or localization of Cet1p and Ceg1p is regulated under certain conditions.

Taken together, we clarified the molecular mechanism and biological significance of nuclear transport of the Cet1p-Ceg1p capping enzyme complex in budding yeast. Our study is the first to demonstrate that the interaction of Cet1p and Ceg1p is critical for both nuclear localization and activity of the mRNA capping enzyme complex *in vivo*. Our data help us to understand the regulation of mRNA capping and to develop new antifungal drugs targeted to the Cet1p-Ceg1p interaction, which plays essential roles in the cell.

## Supporting Information

Figure S1 **Importance of the Cet1p-Ceg1p interaction for yeast cell growth.** (A) Localization of NLS-Ceg1p-GFP in cells expressing Cet1(4A)p. The yeast strain *cet1*Δ*ceg1*Δ was transformed with both *2 μ URA3 CET1* (or *CET1(4A)*) and *CEN HIS3 NLS-CEG1-GFP* plasmids. The cell nucleus was stained with DAPI after fixation. (B) Lethal phenotype of the yeast strain expressing Cet1(4A)p and NLS-Ceg1p-GFP. The indicated strains were streaked on agar plates with or without 0.075% 5-FAA. These plates were incubated at 30°C for 2 days.
(TIF)

Figure S2 **The nuclear transport pathways of Cet1p.** (A) Localization of Cet1(223–227A)p-GFP and Cet1(223–549, 223–227A)p-GFP. The yeast strain *cet1*Δ*ceg1*Δ was transformed with *CEN HIS3 CET1(223–227A)-GFP* or *CET1(223–549, 223–227A)-GFP* plasmid. The cell nucleus was stained with DAPI after fixation. (B) Localization of Cet1p-GFP in kap mutant strains. Each kap mutant strain was transformed with *CEN LEU2 CET1-GFP* plasmid. The cell nucleus was stained with DAPI after fixation. The *ts* strains were grown at 23°C followed by incubation at 37°C for 2 h.
(TIF)

Figure S3 **Complementation of the** ***cet1***Δ***ceg1***Δ **strain by expression of Ceg1p-GFP and Cet1p mutants from high copy plasmids.** The yeast strain *cet1*Δ*ceg1*Δ *CEN HIS3 carrying CEG1-GFP and the 2 μ URA3 CET1* N-terminal deletion mutants or wild type CET1 (all without *CEN TRP1 hCAP1*) were grown in YPD medium and put on agar plates. These plates were incubated at 25, 30, or 37°C for 2 days.
(TIF)

Table S1 **Primers used in this study.**
(DOC)

Table S2 **Plasmids used in this study.**
(DOC)

Table S3 **Yeast strains used in this study.**
(DOC)
