# Supplementary figures and images for: The Essential Role for the RNA Triphosphatase Cet1p in Nuclear Import of the mRNA Capping Enzyme Cet1p-Ceg1p Complex of Saccharomyces cerevisiae
Source: PLoS One. 2013 Oct 30;8(10):e78000. doi: 10.1371/journal.pone.0078000 (PMC3813497; doi:10.1371/journal.pone.0078000)

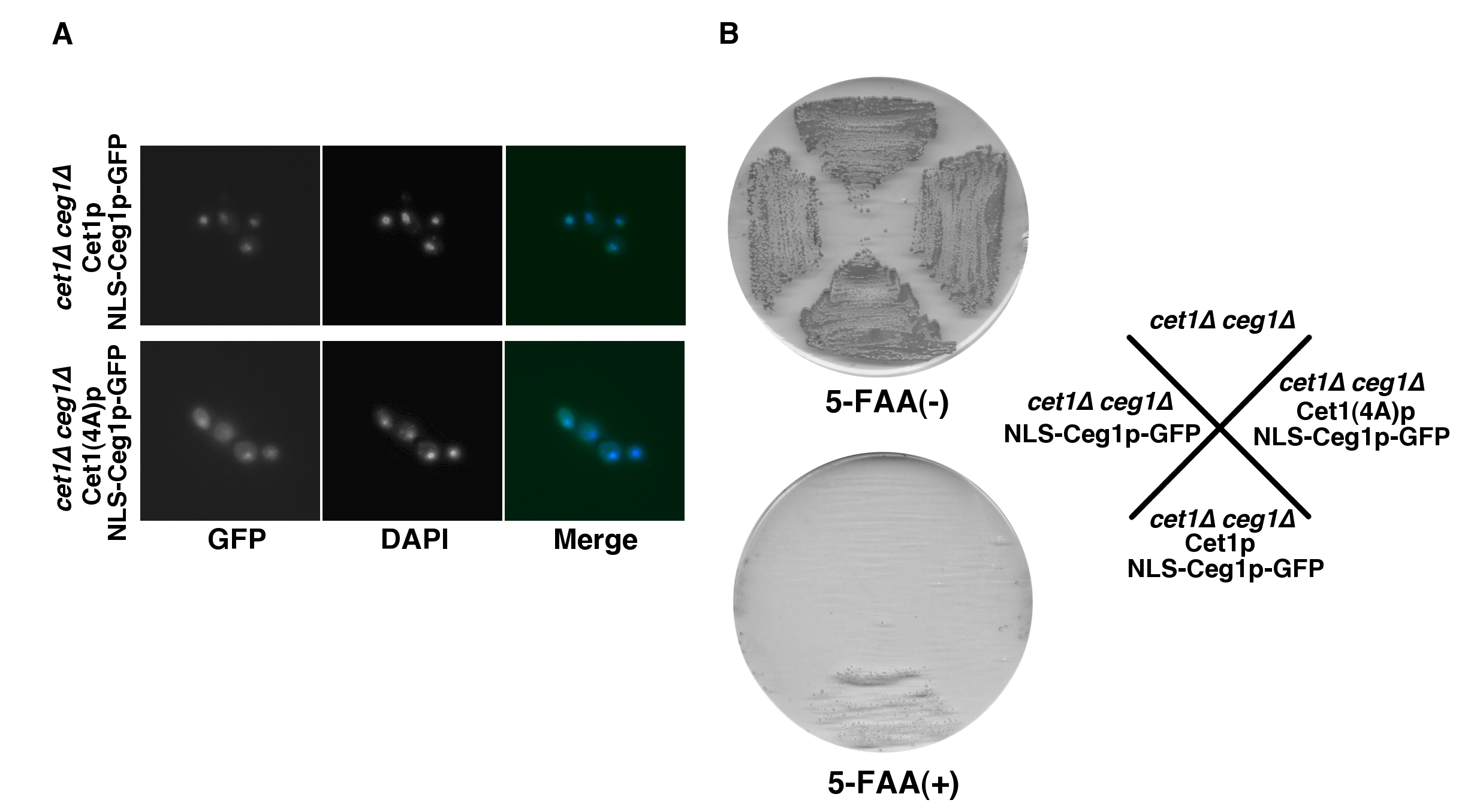

Supplement: Figure S1 — Importance of the Cet1p-Ceg1p interaction for yeast cell growth. (A) Localization of NLS-Ceg1p-GFP in cells expressing Cet1(4A)p. The yeast strain cet1Δceg1Δ was transformed with both 2 μ URA3 CET1 (or CET1(4A)) and CEN HIS3 NLS-CEG1-GFP plasmids. The cell nucleus was stained with DAPI after fixation. (B) Lethal phenotype of the yeast strain expressing Cet1(4A)p and NLS-Ceg1p-GFP. The indicated strains were streaked on agar plates with or without 0.075% 5-FAA. These plates were incubated at 30°C for 2 days. (TIF) [file pone.0078000.s001.tif]

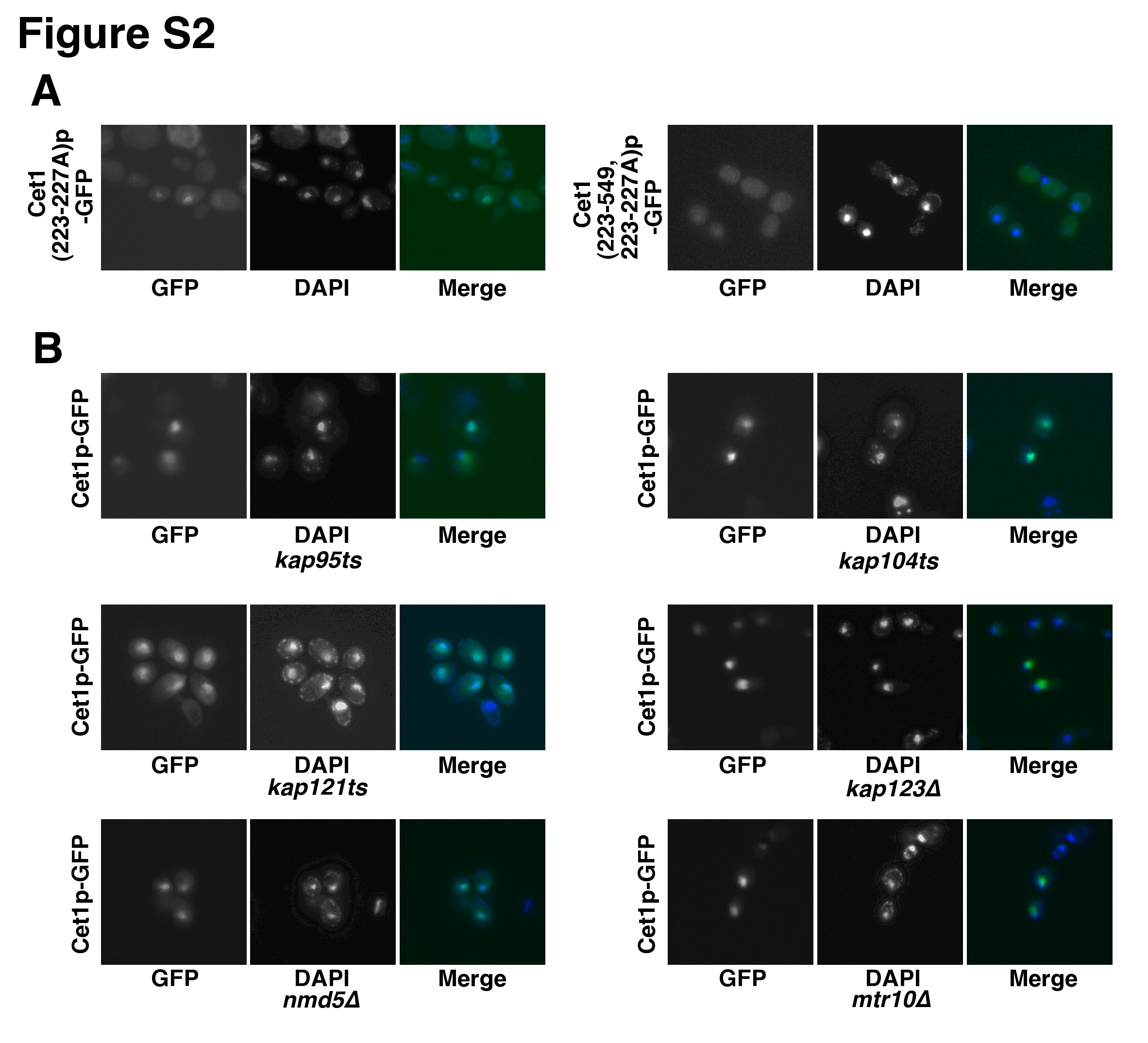

Supplement: Figure S2 — The nuclear transport pathways of Cet1p. (A) Localization of Cet1(223–227A)p-GFP and Cet1(223–549, 223–227A)p-GFP. The yeast strain cet1Δceg1Δ was transformed with CEN HIS3 CET1(223–227A)-GFP or CET1(223–549, 223–227A)-GFP plasmid. The cell nucleus was stained with DAPI after fixation. (B) Localization of Cet1p-GFP in kap mutant strains. Each kap mutant strain was transformed with CEN LEU2 CET1-GFP plasmid. The cell nucleus was stained with DAPI after fixation. The ts strains were grown at 23°C followed by incubation at 37°C for 2 h. (TIF) [file pone.0078000.s002.tif]
