# Supplementary material for: The Essential Role for the RNA Triphosphatase Cet1p in Nuclear Import of the mRNA Capping Enzyme Cet1p-Ceg1p Complex of Saccharomyces cerevisiae
Source: PLoS One. 2013 Oct 30;8(10):e78000. doi: 10.1371/journal.pone.0078000 (PMC3813497; doi:10.1371/journal.pone.0078000)
Supplement: Table S1 — Primers used in this study. (DOC) [file pone.0078000.s004.doc]

Table S1. Primers used in this study.

| **Name** | **Sequence** |
| --- | --- |
| cet1-GFP for | TTTATCATATGAAATTTTTGAAGGTTCAAAGAAAGTCATGCGGATCCCCGGGTTAATTAA |
| cet1-GFP rev | ATTTCTCGCTCAAGGGCATTTGCTTATTTTTTTTTGAAATGAATTCGAGCTCGTTTAAAC |
| ceg1-GFP for | TAAGGAGCCAAAATATGTAGACGAGGATGATTGGTCGGATCGGATCCCCGGGTTAATTAA |
| ceg1-GFP rev | AATTTAGCAAAAGCAATTGCTTTTGCTTTTCTATCTTATTGAATTCGAGCTCGTTTAAAC |
| NotI cet1 promoter for | TTTGCGGCCGCCTTGTGTCCATGTTATGTTACGTTATG |
| ClaI ADH1 ter rev | TTTTATCGATGGTGTGGTCAATAAGAGCGAC |
| NotI ceg1 promoter for | TTTGCGGCCGCCAGCTGAAAGGCAGATGATCTC |
| cet1 delta for | TTCCAAAATATCCCTTATAAATTGAATCTGGAATAGCACTGTTTAGCTTGCCTCGTCCCC |
| cet1 delta rev | CAAGGGCATTTGCTTATTTTTTTTTGAAATGATTCAAATATGGATGGCGGCGTTAGTATC |
| cet1 delta201 for | GCGAATGCAGCTGTAGATAATATTT |
| cet1 delta201 rev | CATAGTGGGAGGATAGAAATGC |
| cet1 delta275 for | ATTATTCCCGATGATGACTTAACAAAGT |
| cet1 4A for | GCTGCTAAACCTACTATCAAAGCTCTTCAA |
| cet1 4A rev | AGCTGCAGCAATTGGAACATTCCTGTACTT |
| cet1 delta218 for | AAAAAGAATAATATCAAGAGAGATTTGG |
| cet1 delta246 for | ATTTGGGCACAAAAATGGAAACCTAC |
| Cet1_223_for | AAGAGAGATTTGGAGGTTCTGAATG |
| Cet1_228_for | GTTCTGAATGAAATATCTGCGTCTTC |
| Cet1_233_for | TCTGCGTCTTCCAAGCCCAG |
| Cet1_238_for | CCCAGTAAGTACAGGAATGTTCC |
| NLS-ceg1 for | AGAAAGGTAGAAGACGTTTTAGCAATGGAAAGTAGAGTG |
| NLS-ceg1 rev | CTTCTTTTTTGGCATATTTGTTAAATTAGCGGTCGATTC |
| Cet1_223_227A for | GCTGCTGCAGTTCTGAATGAAATATCTGCGTC |
| Cet1_223_227A rev | TGCTGCGATATTATTCTTTTTTGATTGTAAAG |
| Cet1_∆223_227A for | GCAGCAGCTGCTGCAGTTCTGAATG |
| Cet1_D307Q for | TTACAAATGAAATTTGGTGTTATTATTGAT |
| Cet1_D305Q rev | CTGAATGAAGGATCTTAGTTCAGGA |
