# Supplementary material for: The Essential Role for the RNA Triphosphatase Cet1p in Nuclear Import of the mRNA Capping Enzyme Cet1p-Ceg1p Complex of Saccharomyces cerevisiae
Source: PLoS One. 2013 Oct 30;8(10):e78000. doi: 10.1371/journal.pone.0078000 (PMC3813497; doi:10.1371/journal.pone.0078000)
Supplement: Table S2 — Plasmids used in this study. (DOC) [file pone.0078000.s005.doc]

Table S2. Plasmids used in this study.

| **Plasmid** | **Description** | **Source** |
| --- | --- | --- |
| pFA6a-GFP(S65T)-KanMX6 | Source of GFP(S65T)-KanMX | Shibagaki *et al.*, (1992) *J Biol Chem*, **267**, 9521 |
| pYGT6 | Episomic, *URA3* marker, *CEG1* promtoer, *CEG1* | Wach *et al.*, (1997) *Yeast*, **13**, 1065 |
| pRS313-Cet1 GFP | Centromeric, *HIS3* marker, *CET1* promoter, *CET1-GFP* | This study |
| pRS313-Ceg1 GFP | Centromeric, *HIS3* marker, *CEG1* promoter, *CEG1-GFP* | This study |
| YEp-Cet1 | Episomic, *URA3* marker, *CET1* promtoer, *CET1* | Tsukamoto *et al.*, (1997), *BBRC*, **239**, 116 |
| YEp-Cet1 4A | Episomic, *URA3* marker, *CET1* promoter, *CET1* | This study |
| pET-Cet1 201 | T7 promoter, His-tagged *CET1(201-549)* | This study |
| pET-Cet1 201 4A | T7 promoter, His-tagged *CET1(201-549, 4A)* | This study |
| pGST-Ceg1 | *E. coli* expression, *GST-CEG1* | This study |
| pRS313-Cet1 4A GFP | Centromeric, *HIS3* marker, *CET1* promoter, *CET1(4A)-GFP* | This study |
| pRS313-Cet1 201 GFP | Centromeric, *HIS3* marker, *CET1* promoter, *CET1(201-549)-GFP* | This study |
| pRS313-Cet1 218 GFP | Centromeric, *HIS3* marker, *CET1* promoter, *CET1(218-549)-GFP* | This study |
| pRS313-Cet1 246 GFP | Centromeric, *HIS3* marker, *CET1* promoter, *CET1(246-549)-GFP* | This study |
| pRS313-Cet1 275 GFP | Centromeric, *HIS3* marker, *CET1* promoter, *CET1(275-549)-GFP* | This study |
| pRS313-Cet1 223 GFP | Centromeric, *HIS3* marker, *CET1* promoter, *CET1(223-549)-GFP* | This study |
| pRS313-Cet1 228 GFP | Centromeric, *HIS3* marker, *CET1* promoter, *CET1(228-549)-GFP* | This study |
| pRS313-Cet1 233 GFP | Centromeric, *HIS3* marker, *CET1* promoter, *CET1(233-549)-GFP* | This study |
| pRS313-Cet1 238 GFP | Centromeric, *HIS3* marker, *CET1* promoter, *CET1(238-549)-GFP* | This study |
| YEp-Cet1 218 | Episomic, URA3 marker, *CET1* promoter, *CET1(218-549)* | This study |
| YEp-Cet1 223 | Episomic, URA3 marker, *CET1* promoter, *CET1(223-549)* | This study |
| YEp-Cet1 228 | Episomic, URA3 marker, *CET1* promoter, *CET1(228-549)* | This study |
| YEp-Cet1 233 | Episomic, URA3 marker, *CET1* promoter, *CET1(233-549)* | This study |
| YEp-Cet1 238 | Episomic, URA3 marker, *CET1* promoter, *CET1(238-549)* | This study |
| YEp-Cet1 246 | Episomic, URA3 marker, *CET1* promoter, *CET1(246-549)* | This study |
| pRS313-NLS-Ceg1 GFP | Centromeric, *HIS3* marker, *CEG1* promoter, *NLS(SV40)-CEG1-GFP* | This study |
| YEp-Cet1(D305,307A) | Episomic, URA3 marker, *CET1* promoter, *CET1(D305,307A)* | This study |
| pRS313-Cet1(D305,307A) GFP | Centromeric, *HIS3* marker, *CET1* promoter, *CET1(D305,307A)-GFP* | This study |
| pRS313-Cet1(223-227A) GFP | Centromeric, *HIS3* marker, *CET1* promoter, *CET1(223-227A)-GFP* | This study |
| pRS313-Cet1(223-549, 223-227A) GFP | Centromeric, *HIS3* marker, *CET1* promoter, *CET1(223-549, 223-227A)-GFP* | This study |
| pRS315-Cet1 GFP | Centromeric, *LEU2* marker, *CET1* promoter, *CET1-GFP* | This study |
