# Supplementary material for: The Essential Role for the RNA Triphosphatase Cet1p in Nuclear Import of the mRNA Capping Enzyme Cet1p-Ceg1p Complex of Saccharomyces cerevisiae
Source: PLoS One. 2013 Oct 30;8(10):e78000. doi: 10.1371/journal.pone.0078000 (PMC3813497; doi:10.1371/journal.pone.0078000)
Supplement: Table S3 — Yeast strains used in this study. (DOC) [file pone.0078000.s006.doc]

**Table S3. Yeast strains used in this study.**

| **Strain** | **Genotype** | **Source** |
| --- | --- | --- |
| HC101 | *MATa, ade2-1, ura3-1, his3-11, trp1-1, leu2-3, 112, can1-100, cet1∆2::LEU2,* YCpW-hCAP1 (*CEN TRP1 hCAP1*) | This study (From Y.S.) |
| HC201 | *MATα, ade2-1, ura3-1, his3-11, trp1-1, leu2-3, 112, can1-100, ceg1∆2::LEU2,* YCpW-hCAP1 | This study (From Y.S.) |
| yNT0013 | *MATa, ade2-1, ura3-1, his3-11, trp1-1, leu2-3, 112, can1-100, cet1∆2::LEU2,* YCpW-hCAP1, pRS313-Cet1 GFP (*CEN HIS3 CET1-GFP*) | This study |
| yNT0014 | *MATα, ade2-1, ura3-1, his3-11, trp1-1, leu2-3, 112, can1-100, ceg1∆2::LEU2,* YCpW-hCAP1, pRS313-Ceg1 GFP | This study |
| yNT0026 | *MATα, ade2-1, ura3-1, his3-11, trp1-1, leu2-3, 112, can1-100, ceg1∆2::LEU2, cet1∆::KanMX6,* YCpW-hCAP1 | This study |
| yNT0035 | *MATα, ade2-1, ura3-1, his3-11, trp1-1, leu2-3, 112, can1-100, ceg1∆2::LEU2, cet1∆::KanMX6,* YCpW-hCAP1, pRS313-Cet1 GFP | This study |
| yNT0036 | *MATα, ade2-1, ura3-1, his3-11, trp1-1, leu2-3, 112, can1-100, ceg1∆2::LEU2, cet1∆::KanMX6,* YCpW-hCAP1, pRS313-Ceg1 GFP | This study |
| yNT0034 | *MATα, ade2-1, ura3-1, his3-11, trp1-1, leu2-3, 112, can1-100, ceg1∆2::LEU2, cet1∆::KanMX6,* YCpW-hCAP1, pRS313-Ceg1 GFP, YEp-Cet1 (*2μ URA3 CET1*) | This study |
| yNT0043 | yNT0034 except YEp-Cet1 4A | This study |
| yNT0033 | *MATa, ade2-1, ura3-1, his3-11, trp1-1, leu2-3, 112, can1-100, cet1∆2::LEU2,* YCpW-hCAP1, pRS313-Cet1 4A GFP | This study |
| yNT0037 | yNT0035 except pRS313-Cet1 201 GFP | This study |
| yNT0038 | yNT0035 except pRS313-Cet1 275 GFP | This study |
| yNT0045 | yNT0035 except pRS313-Cet1 218 GFP | This study |
| yNT0046 | yNT0035 except pRS313-Cet1 246 GFP | This study |
| yNT0068 | yNT0035 except pRS313-Cet1 223 GFP | This study |
| yNT0069 | yNT0035 except pRS313-Cet1 228 GFP | This study |
| yNT0070 | yNT0035 except pRS313-Cet1 233 GFP | This study |
| yNT0071 | yNT0035 except pRS313-Cet1 238 GFP | This study |
| yNT0073 | yNT0034 except YEp-Cet1 218 | This study |
| yNT0074 | yNT0034 except YEp-Cet1 223 | This study |
| yNT0075 | yNT0034 except YEp-Cet1 228 | This study |
| yNT0076 | yNT0034 except YEp-Cet1 246 | This study |
| yNT0077 | yNT0034 except YEp-Cet1 233 | This study |
| yNT0078 | yNT0034 except YEp-Cet1 238 | This study |
| yNT0015 | *MATa, ade2-1, ura3-1, his3-11, trp1-1, leu2-3, 112, can1-100, cet1∆2::LEU2,* pRS313-Cet1GFP | This study |
| yNT0090 | yNT0015 except pRS313-Cet1 218 GFP | This study |
| yNT0091 | yNT0015 except pRS313-Cet1 223 GFP | This study |
| yNT0092 | yNT0015 except pRS313-Cet1 228 GFP | This study |
| yNT0093 | yNT0015 except pRS313-Cet1 233 GFP | This study |
| yNT0094 | yNT0015 except pRS313-Cet1 238 GFP | This study |
| yNT0049 | *MATα, ade2-1, ura3-1, his3-11, trp1-1, leu2-3, 112, can1-100, ceg1∆2::LEU2, cet1∆::KanMX6,* YCpW-hCAP1, pRS313-NLS-Ceg1 GFP | This study |
| yNT0050 | *MATα, ade2-1, ura3-1, his3-11, trp1-1, leu2-3, 112, can1-100, ceg1∆2::LEU2, cet1∆::KanMX6,* YCpW-hCAP1, pRS313-NLS-Ceg1 GFP, YEp-Cet1 | This study |
| yNT0058 | *MATα, ade2-1, ura3-1, his3-11, trp1-1, leu2-3, 112, can1-100, ceg1∆2::LEU2, cet1∆::KanMX6,* YCpW-hCAP1, pRS313-NLS-Ceg1 GFP, YEp-Cet1 4A | This study |
| yNT0099 | *MATα, ade2-1, ura3-1, his3-11, trp1-1, leu2-3, 112, can1-100, ceg1∆2::LEU2, cet1∆::KanMX6,* YCpW-hCAP1, pRS313-Cet1(223-227A) GFP | This study |
| yNT0100 | *MATα, ade2-1, ura3-1, his3-11, trp1-1, leu2-3, 112, can1-100, ceg1∆2::LEU2, cet1∆::KanMX6,* YCpW-hCAP1, pRS313-Cet1(D305,307A) GFP | This study |
| yNT0101 | *MATα, ade2-1, ura3-1, his3-11, trp1-1, leu2-3, 112, can1-100, ceg1∆2::LEU2, cet1∆::KanMX6,* YCpW-hCAP1, pRS313-Ceg1 GFP, YEp-Cet1(D305,307A) | This study |
| yNT0102 | *MATα, ade2-1, ura3-1, his3-11, trp1-1, leu2-3, 112, can1-100, ceg1∆2::LEU2, cet1∆::KanMX6,* YCpW-hCAP1, pRS313-Cet1(223-549, 223-227A) GFP | This study |
| *mtr10::HIS3* | *MATα, ade2, ura3, leu2, trp1, his3, mtr10∆::HIS3* | Senger et al., (1998), *EMBO J.* **17**, 2196 |
| *kap104-16* | *MATa, kap104::HIS3, trp1-1, ura3-52, his3∆200, leu2-3, lys2-1,* pRS314 *kap104-16* | Aitchison et al., (1996) *Science* **274**, 624 |
| PSY967 | *MATα, ura3-52, leu2∆1, his3∆200, kap123∆::HIS3* | Seedorf and Silver, (1997) *PNAS* **94**, 8590 |
| PSY1103 | *MATα, ura3-52, leu2∆1, trp1∆63, rsl1-4* | Ferrigno et al., (1998) *EMBO J.* **17**, 2196 |
| PSY1199 | *MATα, ade2∆::hisG, ade8∆100::KAN^R^ ura3∆, leu2∆1, his3∆200, nmd5∆::HIS3* | Ferrigno et al., (1998) *EMBO J.* **17**, 2196 |
| PSY1201 | *MATa, ura3-52, leu2∆1, trp1∆63, pse1-1* | Seedorf and Silver, (1997) *PNAS* **94**, 8590 |
| yNT0053 | *MATα, ade2, ura3, leu2, trp1, his3, mtr10∆::HIS3, pRS315-Cet1 GFP* | This study |
| yNT0055 | *MATa, kap104::HIS3, trp1-1, ura3-52, his3∆200, leu2-3, lys2-1,* pRS314 *kap104-16, pRS315-Cet1 GFP* | This study |
| yNT0051 | *MATα, ura3-52, leu2∆1, his3∆200, kap123∆::HIS3, pRS315-Cet1 GFP* | This study |
| yNT0054 | *MATα, ura3-52, leu2∆1, trp1∆63, rsl1-4, pRS315-Cet1 GFP* | This study |
| yNT0052 | *MATα, ade2∆::hisG, ade8∆100::KAN^R^ ura3∆, leu2∆1, his3∆200, nmd5∆::HIS3, pRS315-Cet1 GFP* | This study |
| yNT0056 | *MATa, ura3-52, leu2∆1, trp1∆63, pse1-1, pRS315-Cet1 GFP* | This study |
